# How regularity representations of short sound patterns that are based on relative or absolute pitch information establish over time: An EEG study

**DOI:** 10.1371/journal.pone.0176981

**Published:** 2017-05-04

**Authors:** Maria Bader, Erich Schröger, Sabine Grimm

**Affiliations:** Institute of Psychology, Leipzig University, Leipzig, Germany; Aarhus Universitet, DENMARK

## Abstract

The recognition of sound patterns in speech or music (e.g., a melody that is played in different keys) requires knowledge about pitch relations between successive sounds. We investigated the formation of regularity representations for sound patterns in an event-related potential (ERP) study. A pattern, which consisted of six concatenated 50 ms tone segments differing in fundamental frequency, was presented 1, 2, 3, 6, or 12 times and then replaced by another pattern by randomly changing the pitch of the tonal segments (roving standard paradigm). In an absolute repetition condition, patterns were repeated identically, whereas in a transposed condition, only the pitch relations of the tonal segments of the patterns were repeated, while the entire patterns were shifted up or down in pitch. During ERP measurement participants were not informed about the pattern repetition rule, but were instructed to discriminate rarely occurring targets of lower or higher sound intensity. EPRs for pattern changes (mismatch negativity, MMN; and P3a) and for pattern repetitions (repetition positivity, RP) revealed that the auditory system is able to rapidly extract regularities from unfamiliar complex sound patterns even when absolute pitch varies. Yet, enhanced RP and P3a amplitudes, and improved behavioral performance measured in a post-hoc test, in the absolute as compared with the transposed condition suggest that it is more difficult to encode patterns without absolute pitch information. This is explained by dissociable processing of standards and deviants as well as a back propagation mechanism to early sensory processing stages, which is effective after less repetitions of a standard stimulus for absolute pitch.

## Introduction

Meaningful units in speech and music are typically characterized by the relative composition of certain acoustic features. For instance, in speech, it is the relative values of the formants that define individual vowels, or in music, it is the proportional pitch relations between single notes that are among other factors crucial to identify a melodic theme. Absolute features of such units might differ significantly without hampering their identifiability and categorization, for example speech units can be recognized despite a high variability regarding their absolute spectral features both within and between speakers, such as low or high voice pitch. In fact, even when acquiring knowledge about unfamiliar sound sources, learning mechanisms must be able to tolerate such variability. In the current study, we explored the role of absolute and relative pitch information during the initial perceptual learning of complex auditory melodic patterns.

Certainly, pitch itself plays an important role in extracting and learning sound patterns [1,2]. Representations of the exact spectrotemporal properties of a stimulus help segregating auditory objects (i.e. perceptual entities which are perceived as coming from one emitting source [3,4]) from complex auditory scenes [5]. The tonotopic organization of the auditory system with frequency-selective parcellations of the basilar membrane in the inner ear, which are maintained throughout subcortical structures and primary auditory cortex [6,7], suggests a dominant role for absolute frequency coding in auditory processing. This is supported by studies showing sensitivity to frequency changes even outside the attentional focus [8] and for slight frequency changes in complex sound patterns [9].

Nevertheless, in music recognition and speech prosody relative pitch supposedly plays a more important role than absolute pitch [10]. A melody retains its identity despite transposition [11,12] and absolute pitch is mostly disregarded when keeping content in long-term memory [13]. Absolute pitch ability is a rare phenomenon [14,15] and most people don’t show awareness of discrete and isolated pitch information. It has been shown that infants do not prefer the specific mode in which they got familiar with a melody, meaning that relative pitch changes are either not salient for them or they are not able to remember the absolute pitch [16]. Brain imaging studies have also shown that abstract regularities are processed on early stages and independently of absolute pitch [12].

A paradigm suitable to study the formation of a sensory memory trace is the roving standard paradigm, in which a train of stimuli of the same type is eventually interrupted by a different stimulus, which is then repeated in a new stimulus train [17–20]. Responses to stimulus repetitions and stimulus changes can be evaluated as a function of the number of previous repetitions [17,21,22]. Stimulus changes elicit typical event-related potentials (ERPs), such as the mismatch negativity (MMN) [23] and the P3a component of the ERP [24,25]. MMN appears 100 to 250 ms post stimulus and reflects an automatic and pre-attentive auditory change-detection mechanism [23]. Since its presence implies that a regularity representation of the preceding regular stimuli has been established, it is interpreted as an indirect marker for regularity extraction and deviance detection. Its amplitude increases with the number of repeated standards [17,21,22,26–28] reflecting the growing strength of the regularity representation. For simple rules and simply structured stimuli, MMN occurs after only one exact repetition of a standard sound, implying that a regularity representation is rapidly established [17,26]. For more abstract rules like feature relations it takes at least three [26] or even more presentations of the standard sound before a regularity representation is formed [26,29]. Whereas previous studies have shown, that MMN is also elicited for changes in complex spectrotemporal stimuli [30–33], the time course with which reoccurring dynamically structured stimuli are memorized has not been investigated yet.

Additionally, stimulus changes often elicit the P3a, which consists in a large positive-going deflection following MMN [24,25]. P3a is associated with the detection of a distracting sound within a stream of matching sounds and a possible subsequent shift of orientation or attention towards the acoustic change or novel sound [23,34–36]. A P3a might also be elicited by sudden sounds breaking through a silent environment where the novel event has captured involuntarily the focus of attention [37,38]. Therefore P3a appears to reflect an aspect of stimulus evaluation and orienting response [25,39], which habituates rapidly within the first few repetitions of an initial deviating stimulus [25,40–42].

Whereas MMN and P3a are associated with the processing of stimulus changes, the roving standard paradigm additionally allows the investigation of direct effects of stimulus repetition. With higher numbers of repetitions, an increasing positivity emerges in the stimulus ERP in the time range from 50 to 200 ms, the so-called repetition positivity (RP), which has been interpreted as a more direct marker of sensory memory trace formation [43,44]. Baldeweg and colleagues explain RP effects by a back-propagation mechanism of auditory memory traces from higher to lower sensory levels with increasing trace strength. As a stimulus is repeated, backward projections enable the suppression of a prediction error, which shows up as a repetition effect on early processing stages [21,43,45]. RP has been robustly observed using simply structured stimuli and sequences, e.g. sine waves roving in tone frequency [22,44]. However, Bendixen and colleagues did not observe RP with a slightly more variable sequence [26]. Nevertheless, its late part (coinciding with the auditory P2 and MMN) has been shown to be unaffected by variance in the sequence [46]. Whether complex dynamic auditory stimuli presented in a roving standard paradigm are suitable to elicit RP has not been shown, yet.

The aim of the current study was to examine implicit regularity encoding of unfamiliar complex sound patterns relying either on absolute pitch information or on relative pitch information alone. In an absolute repetition condition (abs), sound patterns were presented in a roving standard paradigm using pattern repetitions without physical variation. In a transposed repetition condition (trans), only the pitch relations of a pattern were repeated, whereas the pattern could be shifted up or down in pitch; as it happens for example in transposed melodies [47]. If pattern learning occurs rapidly for complex sound patterns, few repetitions should be enough to elicit components associated with regularity violations like the MMN and the P3a. Their amplitudes should increase with increasing numbers of preceding pattern repetitions. Evaluating the particular contributions of pattern change responses and pattern repetition responses (associated with increasing negativity/positivity to the MMN/P3a difference waveform, respectively) will potentially allow for a differentiation between processes of regularity encoding and change detection in the two conditions.

If relative pitch information is sufficient to form regularity representations for complex sound patterns, similar amplitudes and time courses of the emergence of MMN and RP are to be expected–which should probably translate into a similar orienting response to pattern changes (P3a) and similar behavioral performance levels in an active pattern change detection task in both conditions. If absolute pitch plays a major role in complex pattern coding, amplitudes of MMN and RP will be reduced (or even absent) pointing to weaker (or even absent) regularity representations. This might eventually be mirrored on a behavioral level in the active deviance detection task in decreased performance levels. If both absolute and relative pitch information play a role in the emergence of pattern regularity representations–resulting in gradual rather than all-or-none modulations of the targeted components–the paradigm is potentially able to distinguish between modulations of representation strength (amplitude differences) and modulations of the time course of emergence (e.g., more repetitions needed to elicit MMN or RP).

## Materials and methods

### Participants

The experimental protocol was approved by the Ethical Committee of the Leipzig University. Participants gave written informed consent before the experimental session in accordance with the Code of Ethics of the World Medical Association (Declaration of Helsinki). All subjects in the experiments participated for credit points or payment (6 € per hour) and reported normal hearing. Twenty-one healthy subjects (19–44 years, 14 female) participated in this experiment. Assessed by self-report participants had on average *M* = 5.11 years (*SD* = 5.70, *Min* = 0, *Max* = 20) experience with musical instruments. Five of them reported to have no experience in musical training. None of them was a professional musician. During the time of data collection for this study four subjects stated to be active with a musical instrument in their free time. Due to a low signal-to-noise ratio and excessive EEG artifacts three participants had to be excluded from data analyses.

### Stimuli and design

**Fig 1:**
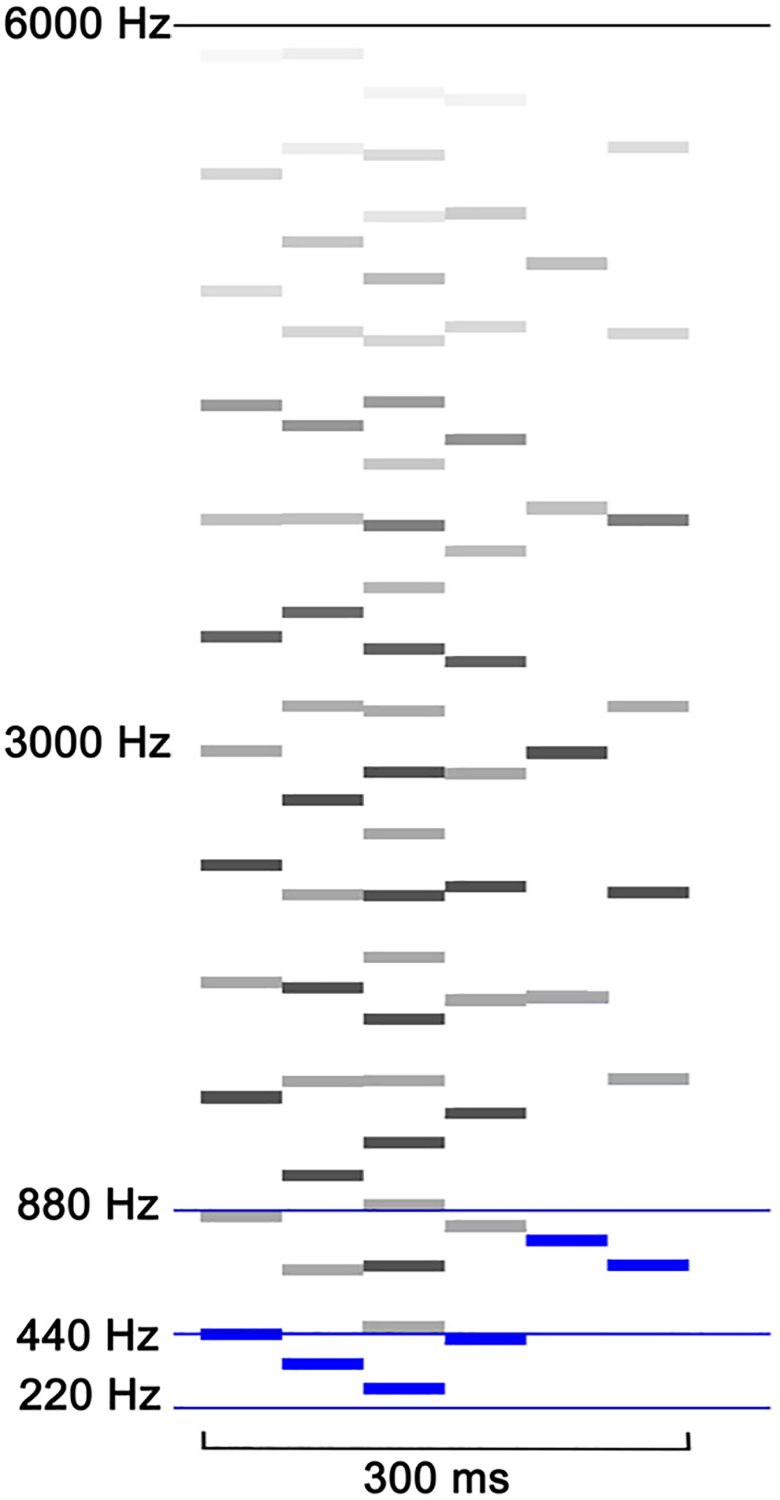
Visualization of sound pattern composition. Bars indicate fundamentals (blue) and harmonics (grey). Lighter grey bars indicate amplitude reduction of uneven harmonics of 20% and a linear slope above 3000 Hz respectively.

Auditory stimuli were 300 ms sound patterns consisting of six 50 ms segments with each segment’s fundamental frequency being randomly chosen from a pool of semitones between 220 and 880 Hz (2 octaves). Harmonics were added to the fundamental frequency until the cutoff at 6000 Hz. Starting at 3000 Hz, harmonics were attenuated by sloping the signal linearly resulting in 0% intensity at 6000 Hz. For a smoother sound uneven harmonics were additionally attenuated to 20% of their intensity (Fig 1).

Segments included a 5 ms rise and a 5 ms fall time. To minimize intensity differences due to potential different numbers of harmonics, segments were root mean square adjusted.

Sound patterns were presented in a roving paradigm [17–19,48] with varying train-lengths, that is within each train the same sound pattern was presented 1, 2, 3, 6, or 12 times and was followed by a train of a different randomly generated sound pattern (see Table 1). The first sound pattern of each new train served as the deviant and the last stimulus of the preceding train as the standard [44]. Since we were interested in memory trace formation starting with the first repetition of a pattern, we included a train-length 1, containing pattern changes that do not follow a pattern repetition. This serves as a neutral reference against which the effects obtained for the other train-lengths can be compared. To gather stimuli for the train-length 1, always three pattern changes occurred in a row, from which the first one served as a deviant with respect to the previous train, the second served as a “standard” of train-length 1 and the third served as a “deviant” of train-length 1. Please note that the terminology here is consistent with the other train-lengths, but arbitrary, since those stimuli do not have an actual history of pattern repetition. Thus for each train-length, a similar amount of standard and deviant patterns was available for ERP analysis.

**Table 1:** Example for the roving standard paradigm.

**pattern**	. . .	A	A	A	A	B	B	C	C	C	D	E	F	F	F	F	F	F	F	F	F	F	**. . .**
**role**	. . .	S_3_	S_4_	S_5_	S_6_	D_6_	S_2_	D_2_	S_2_	S_3_	D_3_	S_1_	D_1_	S_2_	S_3_	S_4_	S_5_	S_6_	S_7_	S_8_	S_9_	S_10_	**. . .**

The upper row shows a possible sound pattern sequence. The occurrence of a new letter (A to F) indicates a pattern change, i.e. the beginning of a new train. The bottom row specifies the role of the sound pattern as being a standard (S) or deviant (D) stimulus in the design. The lower index number refers to the train-length for the deviants, e.g. D_6_ is a deviant after train-length 6. For the standards the lower index indicates the position in the train for the standards, e.g. S_4_ is a standard at the forth position in the train. Only the first and last stimuli of each train (underlined in the lower row) enter the ERP analysis resulting in a similar of amount of stimuli in each condition.

In each block, all possible train-lengths occurred 10 times each in random order, resulting in a sequence of 240 stimuli (50 deviants, 190 standards). The stimulus onset-to-onset interval (SOA) within and between trains was set at 650 ms. Each block had a duration of approximately 3 minutes.

Whereas in the absolute repetition condition (abs), sound patterns were exactly repeated within the trains, in the transposed repetition condition (trans), only the pitch relations of the sound patterns were repeated. That is, the entire pattern was shifted up or down in pitch within one octave choosing a random step out of a pool with 24 half semitone steps. To ensure perceptually distinguishable transpositions, consecutive patterns were transposed with the restriction of at least one semitone.

In order to avoid that in the absolute repetition condition the pattern regularity is extractable based on the pitch of the initial segment alone, in this condition the first segment was kept constant at 440 Hz fundamental frequency. Thus, the earliest time point within a sound pattern at which a pattern change could be detected was in both conditions the onset of the second segment. For each condition, 10 consecutive blocks were presented. Condition order was counterbalanced across participants.

In each block, 10 target stimuli appeared. A target was a single sound pattern (300 ms) from the sequence, which varied in intensity. 5 of the targets were presented with higher volume (+4 dB) and 5 of the targets were presented with lower volume (-4 dB). Targets were distributed randomly over each block with the restriction of at least 2 non-targets in between, at least 5 non-targets at the beginning of each block and at least 2 non-targets at the end of each block.

### Procedure

All experimental procedures were carried out at the Institute of Psychology at Leipzig University. At the beginning of the session, participants performed the Melody part of the Musical Ear Test (MET) [49], which consists of 52 presentations of two short melodies (3 to 8 tones each melody) played with 100 bpm after one another with a sampled piano sound. Participants had to decide by crossing “YES” or “NO” on an answer sheet whether the two melodic phrases were identical or not. The items include contour and interval variations for non-identical melodies. The audio take was presented over headphones (Sennheiser HD 25). This part of the MET lasted 10 minutes.

During the EEG session participants were seated in an electrically shielded chamber. The chamber was sound attenuated and subjects were instructed to fixate a cross on a computer screen placed outside the chamber at a distance of approximately 130 cm. Stimuli were presented binaurally over headphones (Sennheiser HD 25) at an intensity level of 78 dB SPL.

Participants were not informed about the roving rule. While listening to the presented sound patterns and ignoring pattern changes, subjects performed a loudness task by detecting 10 occasionally occurring targets in each block. Participants were instructed to press the left button of a response pad as fast as possible as they were detecting a sound pattern at lower volume and to press the right button of the response pad as they were detecting a sound pattern at higher volume.

After finishing a block in the EEG session, in which subjects were asked to detect rare loudness changes of single sound patterns, subjects got feedback on their performance (hit rate, interchanged buttons, false alarms and their mean reaction time) and had a short break allowing for movements.

As a final part, participants performed an active pattern change detection task to test for the behavioral detectability of pattern changes. The SOA was prolonged to 1100 ms and participants were instructed to detect the onset of a new train, that is a change of a sound pattern, by pressing a button on the response pad. After a short training, each subject performed one block of each condition. Conditions were counterbalanced over participants.

### Data acquisition and analyses

#### Electrophysiological data

EEG data were collected continuously with 64 Ag/AgCl active scalp electrodes positioned according to the international 10–10 system and mounted in a nylon cap. Eye movements were monitored by external electrodes placed above and below the right eye and at the outer canthi of both eyes to yield vertical and horizontal electro-ocular activity (EOG), respectively. As possible offline references additional electrodes were also placed on the tip of the nose and on each mastoid. All electrode signals were sampled at 512 Hz and amplified using a BioSemi Active-Two amplifier (BioSemi, Amsterdam, The Netherlands).

EEG signals were re-referenced offline to the average signal of the mastoids [50,51] and filtered offline using a 0.5 Hz (cutoff) high pass filter (1 Hz transition bandwidth, filter order 1690) and a 35 Hz (cutoff) low pass filter (10 Hz transition bandwidth, filter order 170). Both filters were zero-phase Hamming windowed sinc FIR filters with a stopband attenuation of -54 dB implemented in EEGLab toolbox [52] running under Matlab R2014a (MathWorks, Natick, USA) according to Widmann and colleagues [53]. Epochs were extracted in a window from -100 to 650 ms time-locked to the onset of the stimulus pattern. To avoid the introduction of ongoing activity to the preceding stimulus present in the baseline period into the post-stimulus waveforms, no baseline correction was applied [54]. Sorted averaging was applied for artifact rejection [55]. Trials, in which an intensity target was presented or which were preceded by a target presentation were excluded from analyses. Difference waves were computed by subtracting ERPs to the deviant (dev) sound from those of the corresponding preceding standard (stand) sounds.

#### Statistical analysis

Non-parametrical cluster-based permutation tests were performed with the Fieldtrip MATLAB toolbox [56] to identify significant components of change detection for each condition and to perform a global comparison of conditions. In this analysis, data for train-lengths 2, 3, 6, and 12 were collapsed, whereas ERPs to stimuli of train-length 1 were not included since in the absence of a local history of repetition no deviance-related effects are to be expected. The α-level was set to *p* < .05 for channels and *p* < .05 for clusters. Quantifying the effect at the sample level was conducted by means of dependent samples *t*-tests.1000 permutations were drawn and as the time window the whole epoch (-100 to 650 ms) was chosen.

The MMN and P3a latencies were measured from pattern onset and determined as the relative 50% peak amplitude. They were compared between conditions using the jackknife-based approach [57,58].

For the parametric analyses of RP, mean amplitudes elicited by standards were extracted from 50 to 150 ms after stimulus onset at electrode FCz. A condition (abs, trans) x train-length (1, 2, 3, 6, 12) repeated measures ANOVA for mean standard ERP amplitudes was conducted. If a significant interaction was obtained, post-hoc repeated measures ANOVAs with the factor train-length (1, 2, 3, 6, 12) were conducted for each condition separately.

For the parametric analyses of MMN and P3a 100 ms time windows were chosen in which the cluster-based permutation test yielded the components significant and which contained the maximal component deflections averaged over all participants. Thus, a time window from 166 to 266 ms was chosen for the MMN. Due to differences in topography, a 4-way repeated measures omnibus ANOVA including the factors condition (abs, trans), electrode (Fz, FCz, Cz, CPz, Pz), stimulus type (dev, stand), and train-length (1, 2, 3, 6, 12) was applied for mean amplitudes in the MMN time window.

Due to significant latency differences, the time window for the parametric analyses of the P3a amplitudes ranged from 334 to 434 ms for the absolute repetition condition and from 450 to 550 ms for the transposed repetition condition at electrode FCz, which showed consistently highest P3a amplitudes in the two conditions. A condition (abs, trans) x stimulus-type (stand, dev) x train-length (1, 2, 3, 6, 12) repeated measures omnibus ANOVA for P3a amplitudes was conducted.

If a significant three-way interaction condition x stimulus-type x train-length was obtained for MMN or P3a amplitudes, follow-up repeated-measures ANOVAs with the factors stimulus type (dev, stand) x train-length (1, 2, 3, 6, 12) were calculated separately for each condition to assess the presence of a growing difference between standard and deviant responses with increased train-length (i.e. an interaction stimulus type x train-length). If this was the case, we further report the interaction contrasts for single train-length pairs comparing train-lengths 2, 3, 6, and 12 with single pattern presentations i.e. train-length 1, which was a neutral single pattern presentation, neither acting as a deviant nor having a history of previous repetitions. Interaction contrasts reflect the development of the difference response with increased train-length. Additionally, we report simple repeated-measures contrasts comparing train-lengths 2, 3, 6, and 12 with single pattern presentations i.e. train-length 1 for deviants and standard responses separately, to assess how deviants and standard responses change with increased train-length.

Parametric statistical analyses were run with IBM SPSS version 23.0.0.2 (SPSS Inc., Chicago). The Greenhouse–Geisser correction was applied when the assumption of sphericity was violated (in that case corrected *df*s are reported). For all ANOVAs, partial eta squared *(η*_*p*_^*2*^) served as an estimate of effect size, i.e. the proportion of variance accounted for by the model. For Student’s two-tailed *t*-tests Cohen’s *d* was calculated as an estimate of effect size.

#### Behavioral data

Button presses for the loudness detection task during the EEG sessions and during the active pattern change detection task were analyzed in terms of the signal detection theory index of sensitivity (*d'*) and corrected for avoiding infinite values according to Macmillan & Creelman (1991) [59]. Reaction times were measured by calculating the latency between pattern onset and key press. For the loudness task, response latencies greater than two SOAs (1300 ms) were excluded and for the active behavioral detection task response latencies greater than one SOA of 1100 ms were excluded. For correlations the Pearson correlation coefficient *r* was calculated.

## Results

### Behavioral performance in the loudness change detection task

In the loudness change detection task, targets were discriminated with high accuracy. Averaged sensitivity across participants (*N* = 18) was *d'* = 3.815 (*SD* = 0.527, *Min* = 2.586, *Max* = 4.451) in the absolute repetition and *d'* = 3.838 (*SD* = 0.435, *Min* = 2.810, *Max* = 4.474) in the transposed repetition condition. There were no differences between the absolute and the relative repetition condition as revealed by a Student’s two–tailed *t*–test (*t* (17) = 0.186, *p* = 0.855, *d* = 0.090). The reaction times for the absolute repetition condition were *M* = 600 ms (*SD* = 40 ms, *Min* = 504 ms, *Max* = 651 ms) and for the transposed repetition condition *M* = 695 ms (*SD* = 39 ms, *Min* = 615 ms, *Max* = 757 ms). The reaction times were significantly faster in the absolute repetition condition (*t* (17) = -8.316, *p* < 0.001, *d* = -4.033).

### Behavioral performance in the active pattern change detection task

Averaged sensitivity across participants *(N* = 18) for the absolute repetition condition in the active pattern change detection task was *d'* = 3.061 (*SD* = 0.945, *Min* = 1.363, *Max* = 5.025). The behavioral performance in the transposed repetition condition, *d'* = 1.055 (*SD* = 0.666, *Min* = -0.347, *Max* = 2.379), was significantly lower than the behavioral performance in the absolute repetition condition (*t* (17) = 8.375, *p* < 0.001, *d* = 4.062). On average, reaction times for correctly detected target sounds were *M* = 599 ms (*SD* = 45 ms, *Min* = 504 ms, *Max* = 678 ms) in the absolute repetition condition and *M* = 693 ms (*SD* = 40 ms, *Min* = 609 ms, *Max* = 757 ms) in the transposed repetition condition. The reaction times were significantly faster in the absolute repetition condition (*t* (17) = -7.450, *p* < 0.001, *d* = -3.614).

**Fig 2:**
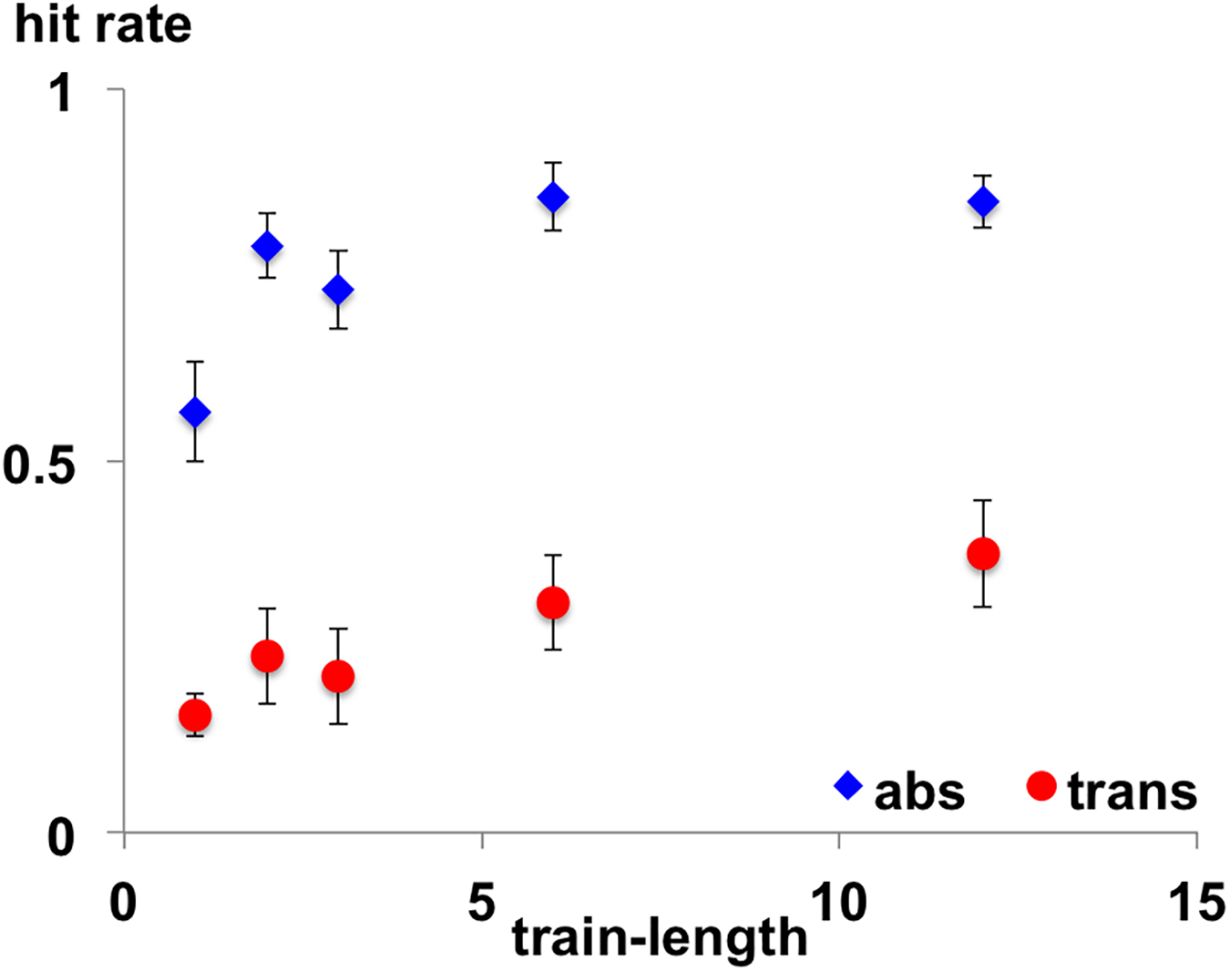
Hit rates in the active behavioral detection task. Proportions of hits (*N* = 18) for the absolute repetition condition (blue triangles) and the transposed repetition condition (red dots) on deviant sound patterns as a function of the number of preceding standard sound patterns are shown. Whiskers indicate standard errors of mean.

Group averaged proportions of hits are presented in Fig 2. A condition (abs, trans) x train-length (1, 2, 3, 6, 12) repeated measures ANOVA of the hit rates showed a significant main effect of condition (*F* (1, 17) = 152.027, *p* < 0.001, *η*_*p*_^*2*^ = 0.899) and a significant main effect of train-length (*F* (2.1, 35.9) = 10.358, *p* < 0.001, *η*_*p*_^*2*^ = 0.379). Contrasts revealed a significant linear trend (*F* (1, 17) = 17.514, *p* = 0.001, *η*_*p*_^*2*^ = 0.507) confirming an increase in the hit rates with increasing train-length. No interaction between the two factors was found (*F* (4, 68) = 0.704, *p* = 0.592, *η*_*p*_^*2*^ = 0.040).

A significant train-length effect was revealed for the absolute repetition condition (*F* (4, 68) = 10.937, *p* < 0.001, *η*_*p*_^*2*^ = 0.391) and contrasts showed, that the hit rates of train-length 1 differed significantly from train-length 2 (*F* (1, 17) = 16.934, *p* = 0.001, *η*_*p*_^*2*^ = 0.499), train-length 3 (*F* (1, 17) = 9.183, *p* = 0.008, *η*_*p*_^*2*^ = 0.351), train-length 6 (*F* (1, 17) = 21.921, *p* < 0.001, *η*_*p*_^*2*^ = 0.563) and train-length 12 (*F* (1, 17) = 18.932, *p* < 0.001, *η*_*p*_^*2*^ = 0.527). A repeated measures ANOVA with the factor train-length revealed only a tendency for a significant effect in the transposed repetition condition: *F* (12.9, 33.1) = 2.650, *p* = 0.087, *η*_*p*_^*2*^ = 0.135. Contrasts did not show significant differences in the proportion of hits between train-lengths 1 and 2 (*F* (1,17) = 2.770, *p* = 0.114, *η*_*p*_^*2*^ = 0.140) and train-length 1 and 3 (*F* (1,17) = 0.683, *p* = 0.420, *η*_*p*_^*2*^ = 0.039), whereas the hit rates of train-length 6 (*F* (1, 17) = 5.575, *p* = 0.030, *η*_*p*_^*2*^ = 0.247) and train-length 12 (*F* (1, 17) = 10.442, *p* = 0.005, *η*_*p*_^*2*^ = 0.381) differed significantly from train-length 1.

#### Correlations of behavioral performance with the MET results

In the MET participants scored on average 75% correct (*SD* = 14%). Results of the MET correlate significantly with the behavioral performance in the absolute repetition condition of the active pattern change detection task: *r* = 0.600, *p* = 0.006. Results of the MET lack a significant correlation with behavioral performance in the transposed repetition condition of the active pattern change detection task: *r* = 0.356, *p* = 0.135. No further correlations between behavioral performance in the active pattern change detection task with latencies or amplitude measures were found.

### EEG data

**Fig 3:**
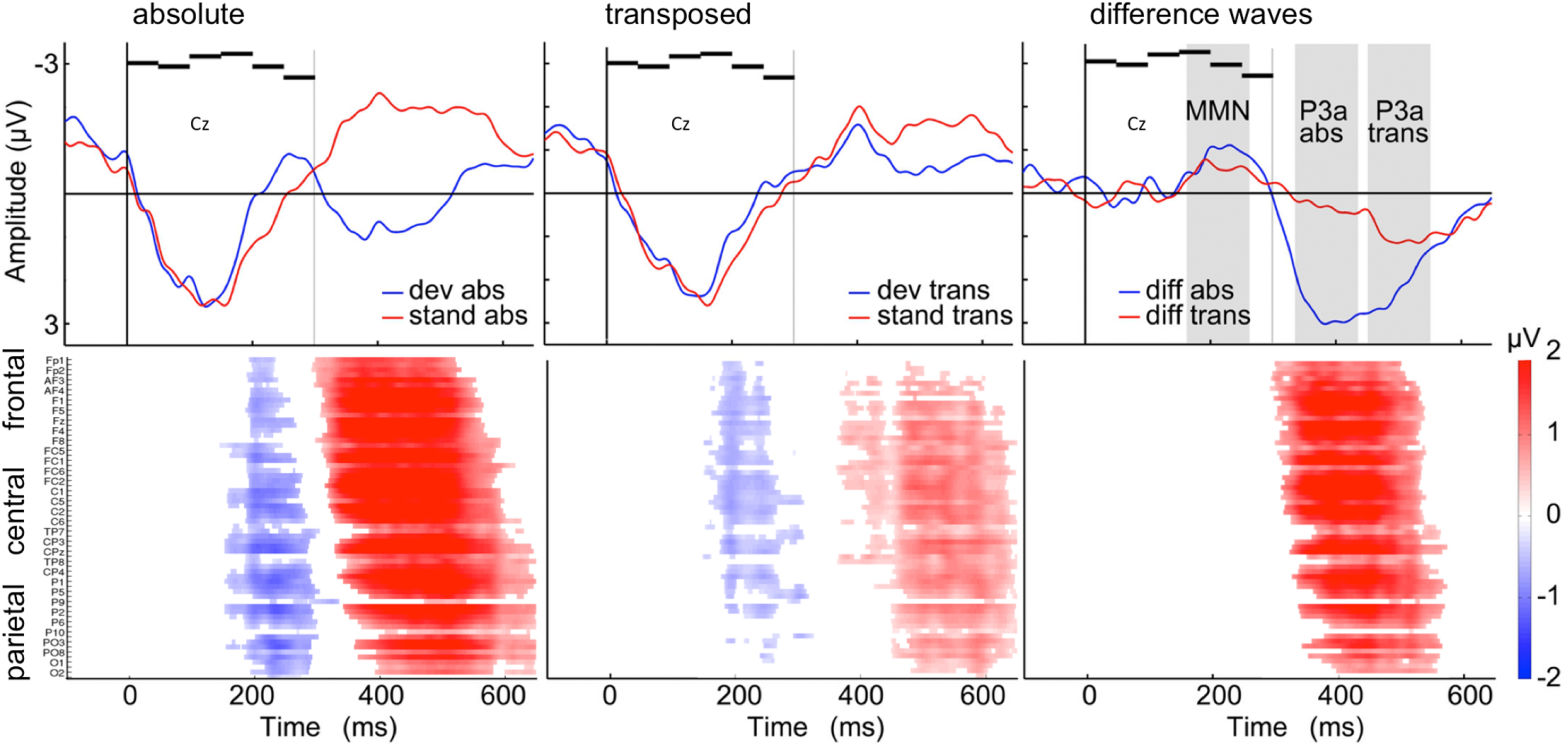
Grand-averaged ERPs, difference waveforms and results of the cluster-based permutation test. *Top*: Grand-averaged (*N* = 18) ERP waveforms at electrode Cz for train-lengths 2, 3, 6, and 12 for deviants (blue) and standards (red) in the absolute repetition condition (left) and transposed repetition condition (middle). Difference waveforms (right) of deviant minus standard are shown for the absolute repetition condition (blue) and for the transposed repetition condition (red). Sound pattern (6 horizontal black bars) onset was at the crossing of the two axes (0 ms) and sound pattern offset was at 300 ms as indicated by the vertical grey line. Please note, that a pattern change could only be detected at the onset of the second segment, that is after 50 ms. Grey shades in the difference wave plots indicate 100 ms time window of MMN and P3a components. *Bottom*: Diagrams illustrating significant differences (*p* < 0.05) between ERPs to deviants and standards from the absolute repetition condition (left) and the transposed repetition condition (middle) according to cluster-based permutation tests. Red and blue portions indicate time points/electrodes in which the ERPs to deviants are more positive and negative, respectively. Color brightness indicates the amplitude of the difference. White portions indicate time points/electrodes at which no significant differences were found. In both conditions, a negative deflection is found around 200 to 300 ms and a positive deflection is found between 350 and 600 ms after stimulus onset. The diagram in the right column shows significant differences between the difference waveforms of the absolute and the transposed repetition condition. Note, that only in the time range of the positive component significantly different component clusters were observed.

**Fig 4:**
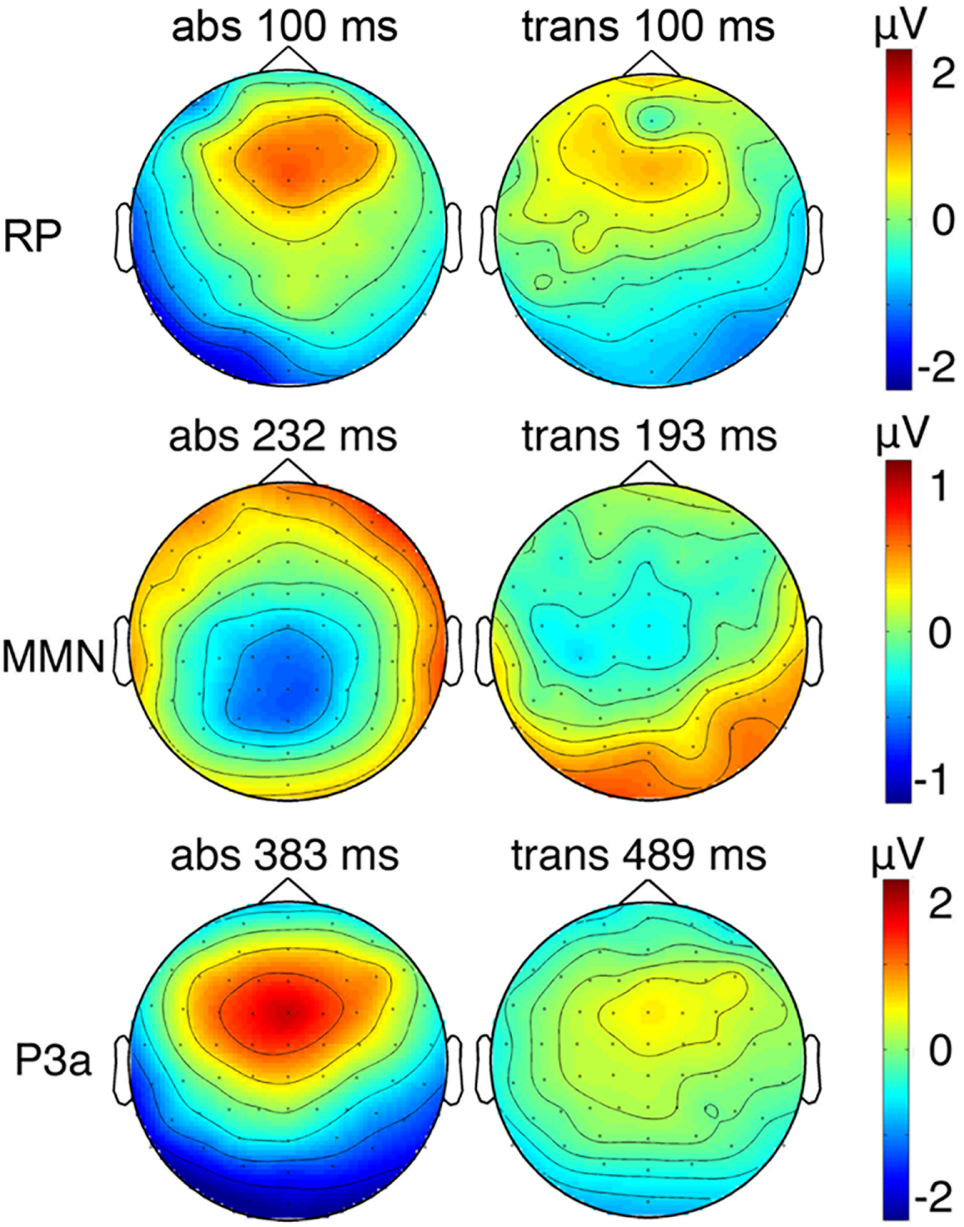
Scalp maps showing RP, MMN and P3a component distribution. Scalp maps are shown at time points with maximal deflections for MMN (top row) and P3a (bottom row) for the absolute repetition condition (abs) and the transposed repetition condition (trans). For RP the differences between train-length 12 and train-length 1 are calculated. Except for the default v4 interpolation between the electrode positions, no additional smoothing (such as a spatial filter) was applied.

**Fig 5:**
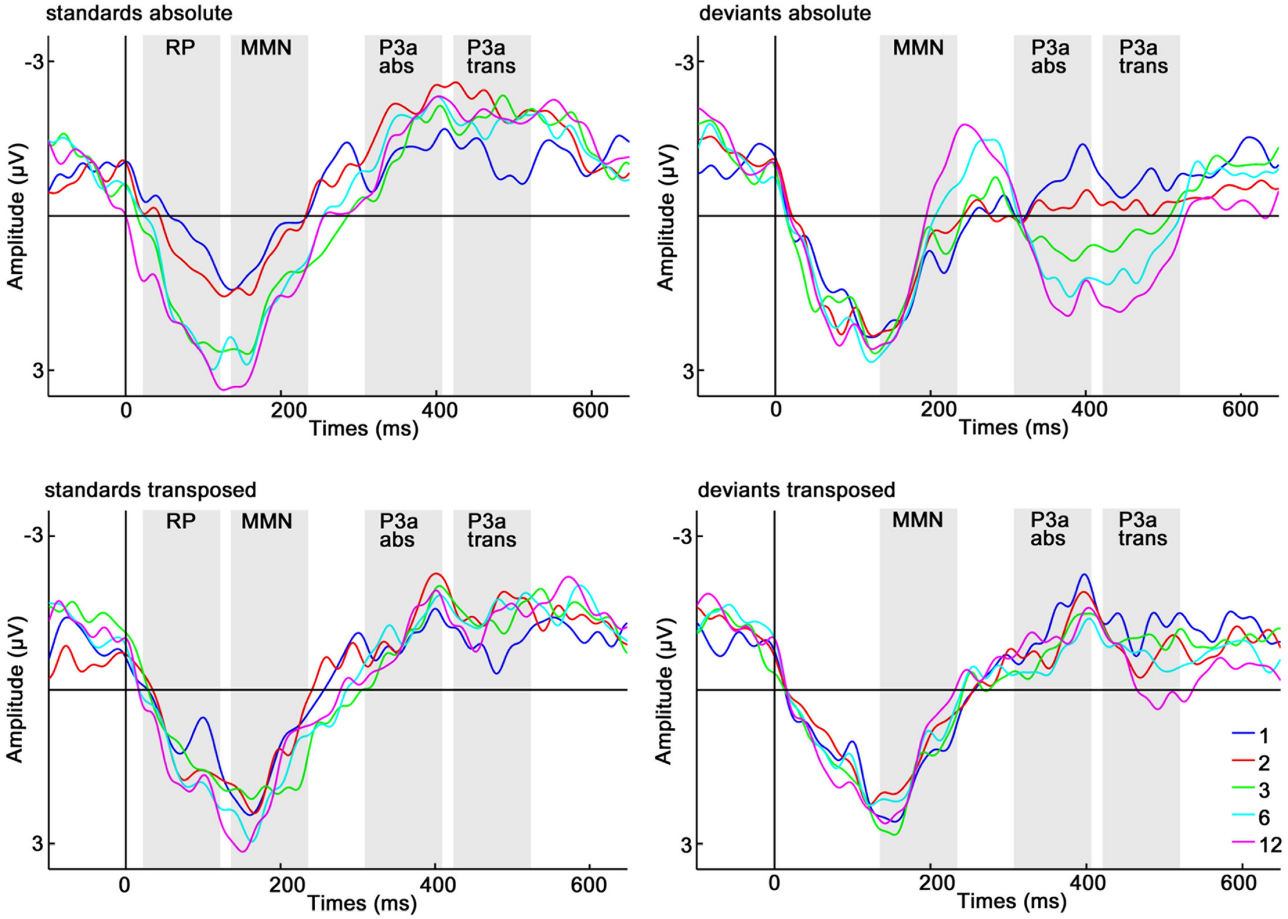
Grand-averaged (*N* = 18) ERPs for single train-lengths. Waveforms of standards at positions 1 (blue), 2 (red), 3 (green), 6 (light blue) and 12 (pink) and of deviants after standard positions 1, 2, 3, 6, and 12 for the absolute (abs) and transposed (trans) repetition condition at electrode Cz are shown. Grey shades indicate 100 ms time windows for RP, MMN and P3a in the absolute and the transposed repetition condition.

Grand averaged difference waveforms (collapsed for train-lengths 2, 3, 6, and 12) elicited in both conditions negative deflections prior to 300 ms after stimulus onset, containing largest contribution from the MMN. In the absolute repetition condition, the strongest negative deflection at midline electrodes was elicited 232 ms after stimulus onset at electrode Pz (*M* = -1.30 μV). Cluster-based permutation tests revealed a time window of significant differences between standard and deviant ERPs ranging from 152 to 289 ms after stimulus onset. Accordingly, for the transposed repetition condition, the strongest negative deflection was found 193 ms after stimulus onset at electrode Cz (*M* = -0.78 μV) and significant differences between standards and deviants occurred in a time window from 166 to 266 ms. The negative component was followed by a larger positive deflection, possibly containing contribution from the P3a, peaking in the absolute repetition condition at 384 ms at electrode FCz (*M* = 3.45 μV) and proving significant in a time window between 313 to 578 ms after stimulus onset. Accordingly for the transposed repetition condition, strongest positive deflection was found 489 ms after stimulus onset at electrode FCz (*M* = 1.26 μV) and occurred within a significant time window of (375 to 443 ms and) 451 to 629 ms. Grand-average waveforms and results of the cluster-based permutations test are shown in Fig 3. Scalp maps of the RP, MMN and P3a component peaks are visualized in Fig 4. Grand-averaged waveforms for standard and deviant responses according to the train-length are visualized in Fig 5.

#### Latency differences

Jackknife estimates of the MMN latency at electrode Cz of relative 50% peak amplitude criteria of the grand-averaged difference waves were *M* = 188 ms (*SD* = 1.87 ms) for the absolute repetition condition and *M* = 171 ms (*SD* = 3.15 ms) for the transposed repetition condition. A Student’s *t*-test did not reveal significant latency differences between conditions (*t* (17)adj = 1.567, *p* = 0.135, *d* = 0.760). Similarly, no significant latency difference was observed at electrode Pz (abs: *M* = 175 ms, *SD* = 10.40 ms; trans: *M* = 186 ms, *SD* = 1.66 ms; *t* (17)adj = -0.288, *p* = 0.777, *d* = -0.140).

Jackknife estimates of the P3a latency at electrode FCz of relative 50% peak amplitude criteria of the difference were *M* = 332 ms (*SD* = 2.07 ms) for the absolute repetition condition and *M* = 463 ms (*SD* = 1.63 ms) for the transposed repetition condition. Here, a Student’s *t*-test revealed highly significant latency differences between conditions (*t* (17)adj = -14.143, *p* < 0.001, *d* = -6.860).

#### RM ANOVA RP

**Fig 6:**
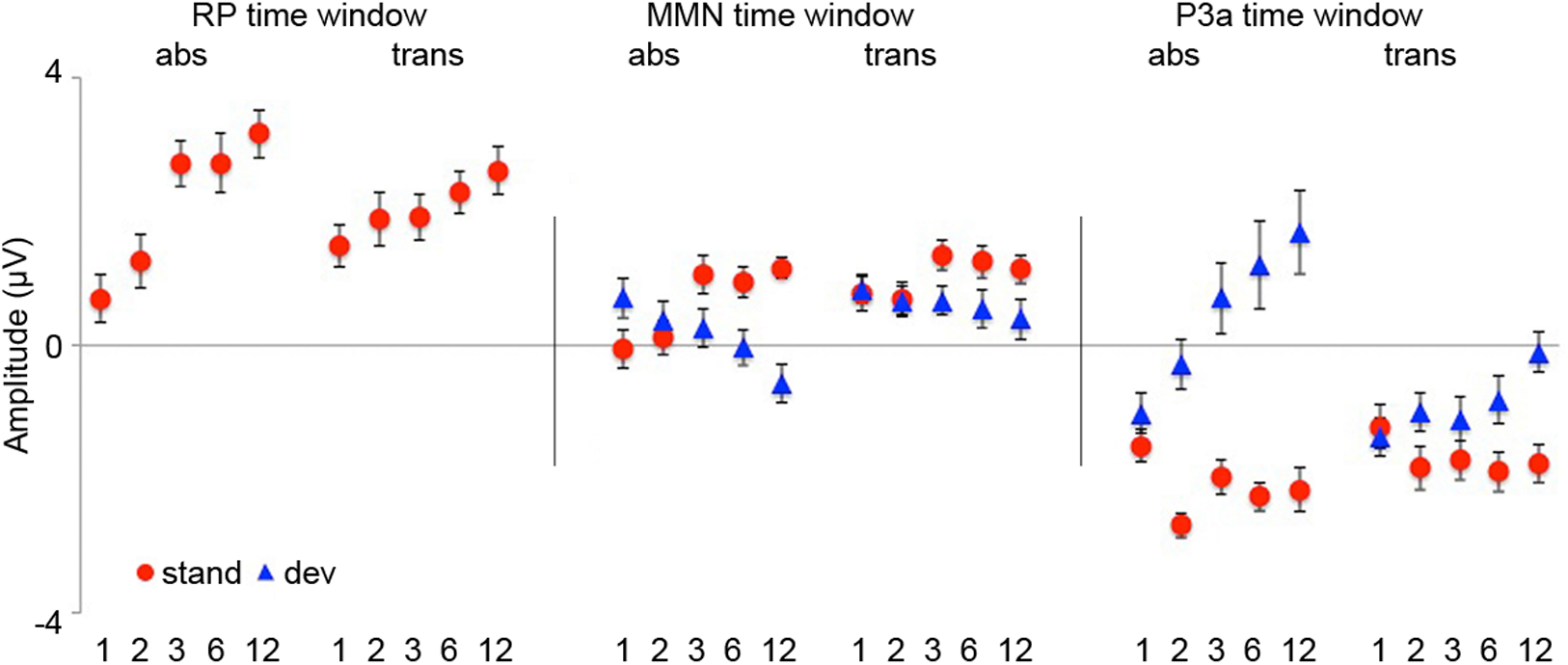
Mean amplitudes for standards and deviants in RP, MMN and P3a time windows. Mean amplitudes (*N* = 18) in 100 ms time windows for RP (50 to 150 ms), MMN (166 to 266 ms) and P3a peak in the absolute (abs: 334 to 434 ms) and in the transposed (trans: 450 to 550 ms) repetition condition elicited to standard (red circles) and deviant (blue triangles) stimuli separately for trains of 1, 2, 3, 6, and 12 sound pattern presentations. Whiskers denote standard errors.

A condition (abs, trans) x train-length (1, 2, 3, 6, 12) repeated measures ANOVA for mean standard ERP amplitudes between 50 and 150 ms after stimulus onset did not reveal a significant main effect of condition: *F* (1, 17) = 0.239, *p* = 0.631, *η*_*p*_^*2*^ = 0.014. The main effect of train-length was highly significant: *F* (4, 68) = 21.735, *p* < 0.001, *η*_*p*_^*2*^ = 0.561, indicating increasing positivity for standards as a function of repetition. This train-length effect was highly linear (*F* (1, 17) = 54.331, *p* < 0.001, *η*_*p*_^*2*^ = 0.762). Also the interaction condition x train-length was significant (*F* (4, 68) = 8.496, *p* < 0.001, *η*_*p*_^*2*^ = 0.333). Post-hoc repeated measures ANOVAs for each condition separately revealed a significant train-length effect for both conditions (see Table 2), but a steeper amplitude increase of standards in the absolute repetition condition, particularly from train-length 2 to 3. In the transposed repetition condition, RP amplitudes started to differ significantly from train-length 1 only at train-length 6, whereas in the absolute repetition condition, RP amplitudes differed significantly from train-length 1 at train-length 2 and all following train-lengths (see Table 2 and Fig 6).

**Table 2:** Train-length (1, 2, 3, 6, 12) effects and contrasts of 2 repeated measures ANOVAs for RP.

	RP					
	stand abs			stand trans		
	*F*	*p*	*η*_*p*_^*2*^	*F*	*p*	*η*_*p*_^*2*^
**train-length**	24.306	0.000	0.588	5.451	0.001	0.243
**1 vs. 2**	4.519	0.048	0.210	1.829	0.194	0.097
**1 vs. 3**	48.341	0.000	0.740	1.910	0.185	0.101
**1 vs. 6**	36.224	0.000	0.681	9.923	0.006	0.369
**1 vs. 12**	58.150	0.000	0.774	16.781	0.001	0.497

RP effects for standard stimulus responses (stand) at electrode FCz in RP time window (50–150 ms after stimulus onset) for the absolute repetition condition (abs) and the transposed repetition condition (trans).

#### RM ANOVA MMN

A condition (abs, trans) x electrode (Fz, FCz, Cz, CPz, Pz) x stimulus type (dev, stand) x train-length (1, 2, 3, 6, 12) repeated measures ANOVA for mean MMN amplitudes (166 to 266 ms after stimulus onset) revealed a significant main effect of stimulus type (*F* (1, 17) = 17.682, *p* = 0.001, *η*_*p*_^*2*^ = 0.510), indicating more negative going amplitudes for deviants than for standards. There also was a main effect of condition (*F* (1, 17) = 39.153, *p* < 0.001, *η*_*p*_^*2*^ = 0.697), resulting from overall more positive amplitudes in the transposed condition. Further, as expected the interaction stimulus type x train-length was significant (*F* (4, 68) = 16.529, *p* < 0.001, *η*_*p*_^*2*^ = 0.493), indicating that differences between deviant and standard amplitudes were growing with increasing number of standard repetitions. The interaction condition x stimulus type was not significant (*F* (1, 17) = 0.219, *p* = 0.645, *η*_*p*_^*2*^ = 0.013).

However, the main effects and the interaction were qualified by an additional condition x stimulus type x train-length interaction (*F* (4, 68) = 3.527, *p* = 0.011, *η*_*p*_^*2*^ = 0.172). Further, the interaction condition x stimulus type x electrode was almost reaching significance (*F* (1.3, 22.7) = 3.658, *p* = 0.058, *η*_*p*_^*2*^ = 0.177), which could be explained by the topographical differences between the difference waveforms in the two conditions (see Fig 4). All further main effects or interactions were not significant (*F* < 2.872, *p* > 0.100).

As there were no significant effects including the factor electrode, amplitude measures were collapsed from all 5 electrodes. Two separate stimulus type (dev, stand) x train-length (1, 2, 3, 6, 12) repeated measures ANOVAs could not explain the three-way-interaction condition x stimulus type x train-length, as they revealed a significant main effect of stimulus type (abs: *F* (1, 17) = 8.443, *p* = 0.010, *η*_*p*_^*2*^ = 0.332, trans: *F* (1, 17) = 19.997, *p* < 0.001, *η*_*p*_^*2*^ = 0.541) and a significant interaction (abs: *F* (4, 68) = 16.618, *p* < 0.001, *η*_*p*_^*2*^ = 0.494, trans: *F* (4, 68) = 2.891, *p* = 0.028, *η*_*p*_^*2*^ = 0.145) in both conditions. In both conditions the stimulus x train-length interaction resulted from larger deviant minus standard differences at train-lengths 3, 6, and 12 than at train-length 1, whereas the deviant minus standard difference was similar at train-length 1 and 2, pointing to significant MMN deflection after 3 standard pattern repetitions (see Table 3).

**Table 3:** MMN and P3a results of 2 repeated measures ANOVA interaction contrasts.

	**MMN**					
	**dev–stand abs**			**dev–stand trans**		
	***F***	***p***	*η*_*p*_^*2*^	***F***	***p***	*η*_*p*_^*2*^
**type x train-length (linear)**	57.109	0.000	0.771	8.561	0.009	0.335
**1 vs. 2**	2.469	0.135	0.127	0.074	0.788	0.004
**1 vs. 3**	20.525	0.000	0.547	11.252	0.004	0.398
**1 vs. 6**	26.556	0.000	0.61	5.120	0.037	0.231
**1 vs. 12**	64.84	0.000	0.792	6.393	0.022	0.273
	**P3a**					
**type x train-length (linear)**	30.750	0.000	0.644	21.458	0.000	0.558
**1 vs. 2**	28.717	0.000	0.628	15.198	0.001	0.472
**1 vs. 3**	24.905	0.000	0.594	4.597	0.047	0.213
**1 vs. 6**	24.273	0.000	0.588	14.004	0.002	0.452
**1 vs. 12**	31.834	0.000	0.652	21.243	0.000	0.555

Results derive from collapsed electrodes in MMN time window (abs, trans: 166 to 266 ms after stimulus onset) and for electrode FCz in P3a time window (abs: 334 to 434 ms, trans: 450 to 550 ms after stimulus onset) with the factors stimulus type (dev, stand) and train-length (1, 2, 3, 6, 12) for the absolute repetition condition (abs) and the transposed repetition condition (trans).

To further explore the origin of the condition x stimulus type x train-length interaction, we analyzed the train-length effects in each condition and for each stimulus type, separately (see Fig 6). A train-length effect was observed for standards in both conditions and a significant amplitude increase could be observed for train-lengths 3, 6, and 12 in the absolute repetition condition and for train-length 3 in the transposed repetition condition. For deviants a train-length effect was only present in the absolute repetition condition, not however in the transposed repetition condition. In the absolute repetition condition, a significant amplitude increase could be observed for train-lengths 6 and 12 with an additional tendency to a significant amplitude increase for train-length 2, whereas no significant amplitude increase could be observed for the transposed repetition condition. For an overview ANOVA values were collected in Table 4.

**Table 4:** MMN and P3a train-length (1, 2, 3, 6, 12) effects and contrasts of 8 repeated measures ANOVAs.

** **	**MMN**											
	**dev abs**			**dev trans**			**stand abs**			**stand trans**		
** **	***F***	***p***	*η*_*p*_^*2*^	***F***	***p***	*η*_*p*_^*2*^	***F***	***p***	*η*_*p*_^*2*^	***F***	***p***	*η*_*p*_^*2*^
**train-length**	7.883	0.000	0.317	0.858	0.494	0.048	11.535	0.000	0.404	3.303	0.016	0.163
**1 vs. 2**	4.035	0.061	0.192	0.636	0.436	0.036	0.604	0.448	0.034	3.579	0.076	0.174
**1 vs. 3**	2.387	0.141	0.123	1.902	0.186	0.101	21.831	0.000	0.562	6.261	0.023	0.269
**1 vs. 6**	12.382	0.003	0.421	1.374	0.257	0.075	16.436	0.001	0.492	3.029	0.100	0.151
**1 vs. 12**	22.697	0.000	0.572	2.450	0.136	0.126	21.187	0.000	0.555	0.491	0.493	0.028
** **	**P3a**											
**train-length**	15.947	0.000	0.484	9.326	0.000	0.354	6.110	0.000	0.264	3.485	0.012	0.170
**1 vs. 2**	6.851	0.018	0.287	3.216	0.091	0.159	33.093	0.000	0.661	11.703	0.003	0.408
**1 vs. 3**	25.26	0.000	0.598	1.272	0.275	0.070	4.003	0.062	0.191	3.691	0.072	0.178
**1 vs. 6**	22.883	0.000	0.574	4.694	0.045	0.216	7.444	0.014	0.305	15.919	0.001	0.484
**1 vs. 12**	36.189	0.000	0.68	31.863	0.000	0.652	4.390	0.051	0.205	5.068	0.038	0.230

Results derive from collapsed electrodes in MMN time window (abs, trans: 166 to 266 ms after stimulus onset) and for electrode FCz in P3a time window (abs: 334 to 434 ms, trans: 450 to 550 ms after stimulus onset) for the absolute repetition condition (abs) and the transposed repetition condition (trans).

### RM ANOVA P3a

A condition (abs, trans) x stimulus type (dev, stand) x train-length (1, 2, 3, 6, 12) repeated measures ANOVA for mean P3a amplitudes did not reveal a significant main effect of condition (*F* (1, 17) = 1.347, *p* = 0.262, *η*_*p*_^*2*^ = 0.073). A main effect of stimulus type (*F* (1, 17) = 55.662, *p* < 0.001, *η*_*p*_^*2*^ = 0.766) and a main effect of train-length (*F* (4, 68) = 9.907, *p* < 0.001, *η*_*p*_^*2*^ = 0.368) were found. All two-way interactions (condition x train-length, condition x stimulus type, train-length x stimulus type) reached a high significance level (*p* < 0.001). However, the main effects and the two-way interactions were qualified by an additional condition x stimulus type x train-length interaction *F* (2.8, 46.9) = 3.426, *p* = 0.028, *η*_*p*_^*2*^ = 0.168).

Two separate stimulus type (dev, stand) x train-length (1, 2, 3, 6, 12) repeated measures ANOVAs for mean amplitudes at electrode FCz (abs: 334 to 434 ms, trans: 450 to 550 ms) revealed a significant main effect of stimulus type (abs: *F* (1, 17) = 41.147, *p* < 0.001, *η*_*p*_^*2*^ = 0.708, trans: *F* (1, 17) = 32.110, *p* < 0.001, *η*_*p*_^*2*^ = 0.654), a significant train-length effect (abs: *F* (4, 68) = 11.346, *p* < 0.001, *η*_*p*_^*2*^ = 0.400, trans: *F* (4, 68) = 3.627, *p* = 0.010, *η*_*p*_^*2*^ = 0.176) as well as a significant interaction (abs: *F* (4, 68) = 14.638, *p* < 0.001, *η*_*p*_^*2*^ = 0.463, trans: *F* (4, 68) = 9.599, *p* < 0.001, *η*_*p*_^*2*^ = 0.361) in both conditions.

In both conditions the stimulus x train-length interaction resulted from larger deviant minus standard differences at train-lengths 2, 3, 6, and 12 compared with train-length 1 (see Table 3) indicating that P3a was elicited already after 2 standard presentations.

Train-length effects were analyzed for each condition and for each stimulus type separately (see Fig 6). A train-length effect was observed for standards and deviants in both conditions. Amplitudes for the deviants in the absolute repetition condition increased significantly for train-length 2, 3, 6, and 12, whereas deviant amplitudes in the transposed repetition condition differed markedly from train-length 1 only for train-length 6 and 12. A significant amplitude decrease for standards was observed at positions 2 and 6 with an additional tendency to a significant amplitude decrease for train-length 3 and 12 in the absolute repetition condition. In the transposed repetition condition significant differences for standard positions 2, 6, and 12 with an additional tendency to a significant amplitude decrease for train-length 3 were found. For an overview ANOVA values are collected in Table 4.

## Discussion

Using a roving standard paradigm, we investigated, whether regularity extraction and change detection for complex spectrotemporal stimuli relies on absolute pitch information (i.e. the exact repetition of a spectrotemporal pattern) or on relative pitch information, tolerating shifts in absolute pitch as long as pitch relations were kept constant.

As expected, pattern changes compared to pattern repetitions elicited an MMN and a subsequent P3a component, which appeared with their typical time course in both conditions comparable to previous studies using auditory oddball [37,60–63] and roving standard paradigms [18,19,21,26].

### Sensory memory trace formation as indexed by MMN and RP

The MMN component occurred rapidly after three presentations of a chosen pattern in both conditions and differences in amplitude between the conditions could not be detected by the non-parametric test. This principally confirms previous findings showing that pattern changes in a sequence of repeated complex sound patterns elicit an MMN [31,32,64]. Our findings indicate that in the case of complex auditory patterns one more repetition is necessary than in situations, in which a simple sound feature is repeated, where mostly two exemplars have been reported to be sufficient to elicit MMN [18,20,44,65,66]. When regularity extraction refers to higher-order features–for instance when pitch relations between successive tones have to be extracted–previous studies showed that at least three presentations of a standard stimulus are necessary for MMN to emerge [26]. As Bendixen and Schröger [26] argue, during the presentation of three stimuli following a relational regularity, in fact two exemplars of the higher-order feature (i.e. the pitch relation) occur. The current data—revealing MMN after 3 pattern exemplars–therefore rather correspond to those studies on higher-order regularities. Interestingly, when spectrotemporal patterns are repeated in different keys, a memory trace appears to establish as rapidly as for identically repeated patterns. At this point one could assume that relative pitch information is sufficient for sensory learning of unfamiliar complex sound patterns. This would be in line with studies showing that for melodies mainly relative pitch information is stored in memory [16]. On a more abstract level this finding would go along with studies showing that the processes underlying MMN evaluate abstract pitch relations and feature conjunctions [67–69].

The gradual increase of MMN amplitudes as a function of preceding number of standard sound patterns replicates findings of previous studies [20–22,32,44,48]. The increase followed a linear trend, which appeared to a similar degree in both conditions. Given the non-equidistant spacing of the train-lengths used, a near logarithmic build-up curve can be assumed [21,22,44,48]. The growth of MMN amplitude is plausibly explained as a result of an increasing positivity for standard stimuli accompanied by an increasing negativity for deviant stimuli [22,43,44,46,48].

Despite the similarity in MMN amplitudes and latencies from the difference wave, ERP responses to pattern deviants differed characteristically between conditions. In the absolute repetition condition the deviant negativity developed for deviants following more than 3 standard presentations. Thus, the increase in MMN with train-length can be attributed to a modulation of both standard and deviant processing, at least in the absolute repetition condition. In the transposed repetition condition, ERPs to pattern changes did not show a clear modulation of the deviant processing as a function of the number of previous standards, that is none of the deviant responses differed significantly from the response to the control deviant of train-length 1, which had no history of previous pattern repetitions. Standard stimulus responses, in contrast, did not show such clear differences between absolute and transposed repetition condition in the MMN time window. That is, whereas conditions did presumably not differ with regard to the matching responses, the mismatch responses developed firmly only in the absolute pitch condition. Even though this effect was distinct, it was not strong enough to come out at the level of the amplitudes of the difference wave. Nevertheless, differences in MMN topographies hint to different cortical areas involved in deviance detection in the two conditions.

Interestingly, our results suggest, that the standard repetition effect starts to occur at position 3 within a train of repeated patterns, whereas the contribution of deviance detection might only take effect at a subsequent position (i.e. for deviants preceded by at least 3 standards). This provides evidence for the assumption that the time course of deviance detection succeeds that of regularity extraction indicated by the finding that the two processes do not arise concurrently.

Thus, even though regularity extraction, in terms of standard repetition effects, and deviance detection, in terms of deviant processing effects, usually seem two sides of the same coin, they are empirically dissociable processes. Previous studies found similar base effects, e.g. Neuloh & Curio [70] or Saarinen and colleagues [68], who found equally large MMN for tone pairs violating a rule regarding their frequency relation and tone pairs violating a rule regarding the absolute tone frequency–though they did not specify how responses of standards and deviants contribute to the MMN in the two cases. Yet, evidence for a dissociation between regularity processing and deviance detection also comes from Pannese and colleagues showing, that the two processes can be differentially modulated by attention and that the auditory system prioritizes information about regularity over information about change [71].

Despite a similar strength of regularity representation at the level of MMN–in terms of the response to standard stimuli—certainly the transposition of single patterns does not go unnoticed in the transposed condition and could actually be processed in addition to the extracted regularity. This might be comparable to cases, in which deviations in pitch relations elicit MMN [68] and in which variations of single features do not influence the MMN if feature conjunctions like frequency relations are proposed to be reflected by the MMN [67,69].

At this point one could conclude, that the time course of sensory memory trace formation, as indexed by the processing of pattern repetitions at the stage of MMN, might be largely independent of absolute pitch information. This would point to the ability of the auditory system to extract abstract regularities unintentionally and rapidly. Nevertheless, the processing of pattern changes themselves seems attenuated when only relative pitch information defines the regularity.

These differential modulations regarding the processing of standard and deviant patterns can be best understood by looking at an earlier ERP time window. Between 50 to 150 ms after stimulus onset, sound patterns showed increased repetition positivity with increased numbers of standard pattern presentations. This has previously been interpreted as a marker of a sensory memory trace formation [43,44]. In general, rule representations were strengthened by further presentations of rule-confirming events in both conditions. However, besides the preserved pitch relations in both conditions, an earlier and stronger repetition positivity effect was observed in the absolute repetition condition. Within the early 50 to 150 ms of the second identical presentation of a sound pattern, the auditory system is able to recognize unintentionally and automatically this absolute repetition and immediately builds up a memory trace. However, repetition positivity effects in the transposed repetition condition were only found after more repetitions.

When absolute pitch varied, an MMN occurred after three pattern presentations, but only for the 6^th^ occurrence of the same standard pattern a RP became measurable. This could be explained in the framework of the back-propagation hypothesis by Baldeweg [21,43], proposing that auditory memory traces exert effects at lower and lower sensory levels with increasing trace strength. In other words, fewer repetitions are needed to show an effect of repetition at later processing stages (MMN time window), whereas only with a higher number of repetitions early processing stages (RP time window) are affected. If the same auditory pattern occurs with variable absolute pitch, a back-propagation to the earliest levels of sensory processing (RP) might initially be hindered or impaired, whereas a back-propagation occurs fast without variation in absolute pitch.

### The role of absolute and relative pitch code on evaluation processes as indexed by P3a and behavior in an active pattern change detection task

Subsequently to the MMN, a P3a component with fronto-central distribution was elicited in both conditions after two standard stimulus presentations. Systematic repetition-related modulations of amplitudes, as mentioned above for MMN, were also found for P3a. Amplitudes increased as a function of the number of preceding standard stimuli in both conditions [17,21,22,26–28,46]. This is congruent with studies showing that the P300 amplitude for task-irrelevant deviants is increased, if they occur with lower probability [72], since in our study decreased local deviant probability (resulting from longer train-lengths) led to an increase of P3a.

Overall, P3a magnitude might indicate the degree of novelty and constitutes a marker of the evaluation processing of the contextual novelty [25,26]. Even though attention was focused on a rule independent task, standard and deviant stimuli captured involuntarily attention and were evaluated on the basis of their underlying pattern structure.

Interestingly, our experimental manipulation affected P3a most dramatically, showing larger amplitudes and earlier component latencies in the absolute compared to the transposed repetition condition. These latency and amplitude differences mirror the difficulty to distinguish implicitly between standard and deviant sound patterns in a relative pitch code context. P3a latency is discussed as being sensitive to degradation and reduction of stimulus discriminability [73]. Similar modulations were found in previous studies, in which Nikjeh and colleagues found a latency shortening and an amplitude increase going along with a higher degree of deviation in harmonic complexes [74]. Another study from Novitski and colleagues also showed, that P3a amplitude and latency were modulated differently by condition and were correlated highly with behavioral performance [75].

In the current study, condition differences might be mainly affected by the processing of deviating events (comparable to the findings for MMN), whereas contributions of standard stimulus responses to the P3a difference waveform were similar in the two conditions. This is in line with a recent study of Barascud and colleagues showing that humans have ideal-observer-like sensitivity–they can actively detect periodically occurring patterns after only 1.5 cycles, that is, during the course of the first pattern repetition [76]. As a replication of the findings of Bendixen et al. [26], standard stimulus responses in the P3a time window developed inversely to standard stimuli in the MMN time window, where amplitudes showed an increasing positivity with an increasing number of consecutively presented standards.

Deviant responses in the P3a time window reflected earlier and stronger effects of pattern changes on the evaluating system in the absolute compared to the transposed repetition condition. This parallels the behavioral performance in the active pattern change detection task, which was substantially faster and more accurate in the absolute repetition condition. A behavioral improvement for detecting pattern changes defined by relative pitch could only be observed after more than six repetitions of a standard sound pattern, whereas the largest improvement in sensitivity occurred already after two standard presentations in the absolute repetition condition. Sensitivity for loudness changes was not affected by condition, but absolute repetitions seemed to help the subjects to respond faster.

This advantage for an absolute pitch code, might primarily be related to pattern change processing, in particular to the evaluation of saliency and novelty of the deviating events. Assuming that each pattern is stored as a unit, the probability that a change (whatsoever type of variance) occurs in a sequence of patterns is relatively low in the absolute condition, since possible variations are either pattern changes or very rare intensity changes, otherwise patterns are invariant. The probability for a physical change to occur in the transposed condition is 100 percent, either in form of a true pattern change, an intensity change, or in form of a transposition of the original pattern. This could explain why processes of novelty evaluation associated with the pattern deviants are delayed and weaker in the transposed condition, despite the sign that the auditory rule is extracted with a similar time course as in the absolute repetition condition.

Since those evaluation processes are also crucial for intentional pattern change detection, prolonged reaction times in the transposed condition are not surprising. In general, the behavioral performance during the detection of pattern changes seems correlated to the musical abilities of our participants as measured by the melody part of the MET–at least for the detection of pattern changes in the absolute pitch condition.

## Conclusion

To sum up, the formation of sensory memory representation for unfamiliar complex patterns occurs rapidly and independently of absolute pitch as deviance-related components were present after only 2 to 3 pattern presentations. However, absolute pitch information fosters a fast development of repetition effects for standard stimuli at an early processing level, as well as the strength of responses to deviant stimuli at the level of MMN and P3a.

If the same auditory pattern occurs with variable absolute pitch, it takes more repetitions until the earliest levels of sensory processing (RP) are affected. Nevertheless, later stages of stimulus evaluation seem tuned to detect an abstract pattern rule as quickly—and almost as reliably—as an exact pattern repetition rule. This could indicate that the memory trace after few repetitions is not as firmly established as needed for similar expectations or predictions on a forthcoming event, as it might be the case for the absolute pitch code.

Consequently, the auditory system is able to rapidly extract regularities from unfamiliar complex sound patterns even when absolute pitch varies. Yet, it seems more difficult to identify pattern changes without additional absolute pitch information if the brain can only rely on pitch relations. This could be explained by the dissociable processing of standards and deviants as well as a back propagation mechanism to early sensory processing stages, which might be effective after less repetitions of a standard stimulus for absolute pitch.

## Supporting information

S1 Data Excel dataset including all behavioral and EEG data that were used for analyses. (XLSX)
